# ATP release and purinergic signaling in NLRP3 inflammasome activation

**DOI:** 10.3389/fimmu.2012.00414

**Published:** 2013-01-08

**Authors:** Aurélie Gombault, Ludivine Baron, Isabelle Couillin

**Affiliations:** Experimental and Molecular Immunology and Neurogenetics, CNRS UMR 7355, University of OrleansOrleans, France

**Keywords:** ATP, danger signal, inflammasome, P2R, NLRP3, purinergic signaling, autophagic cell death

## Abstract

The NLRP3 inflammasome is a protein complex involved in IL-1β and IL-18 processing that senses pathogen- and danger-associated molecular patterns (PAMPs and DAMPs). One step- or two step-models have been proposed to explain the tight regulation of IL-1β production during inflammation. Moreover, cellular stimulation triggers adenosine triphosphate (ATP) release and subsequent activation of purinergic receptors at the cell surface. Importantly some studies have reported roles for extracellular ATP, in NLRP3 inflammasome activation in response to PAMPs and DAMPs. In this mini review, we will discuss the link between active ATP release, purinergic signaling and NLRP3 inflammasome activation. We will focus on the role of autocrine or paracrine ATP export in particle-induced NLRP3 inflammasome activation and discuss how particle activators are competent to induce maturation and secretion of IL-1β through a process that involves, as a first event, extracellular release of endogenous ATP through hemichannel opening, and as a second event, signaling through purinergic receptors that trigger NLRP3 inflammasome activation. Finally, we will review the evidence for ATP as a key pro-inflammatory mediator released by dying cells. In particular we will discuss how cancer cells dying via autophagy trigger ATP-dependent NLRP3 inflammasome activation in the macrophages engulfing them, eliciting an immunogenic response against tumors.

## THE NLRP3 INFLAMMASOME

Innate immunity is triggered by endogenous or environmental danger events through assembly of the NLRP3 inflammasome. The NLRP3 inflammasome is a cytosolic multiprotein platform which is activated in response to a variety of signals including infection, tissue damage, and metabolic dysregulation. Activation of the NLRP3 inflammasome results in the assembly of scaffold components: the cytoplasmic receptor NLRP3, the adaptor protein ASC and the effector protein caspase-1 (Agostini et al., 2004; Martinon et al., 2004, 2009; Martinon and Tschopp, 2004; Kanneganti et al., 2006; Mariathasan et al., 2006). This association leads to the activation of caspase-1, allowing the processing of pro-IL-1β and pro-IL-18 to their mature and secreted forms which are biologically active. IL-1β production is a tightly controlled process playing a pivotal role in inflammation and in recruitment of neutrophils into tissues. A two-signal model has been proposed to explain the regulation of IL-1β production. First the synthesis of pro-IL-1β and NLRP3 is triggered by transcriptional induction via ligands for Toll-like receptors (TLRs), whereas a second stimulus leads to inflammasome oligomerization, caspase-1 auto-activation, caspase-1-dependent cleavage of pro-IL-1β and then release of the biologically active, mature IL-1β. This second signal may be induced by a broad variety of chemically and biologically unrelated molecules classified as either pathogen-associated molecular patterns (PAMPs) or danger-associated molecular patterns (DAMPs). DAMPs originate from environmental pollutants including silica and asbestos (Dostert et al., 2008; Halle et al., 2008), from vaccines such as aluminum salt (alum) adjuvant (Eisenbarth et al., 2008) or from endogenous metabolic stresses such as high concentration of glucose (Schroder et al., 2010), cholesterol (Duewell et al., 2010), amyloid-β protein (Halle et al., 2008), biglycan (Babelova et al., 2009), adenosine triphosphate (ATP; Mariathasan et al., 2006), or monosodium urate (MSU) crystals (Aganna et al., 2002; Eisenbarth et al., 2008). Two major upstream mechanisms are currently proposed for NLRP3 inflammasome activation: plasma membrane disruption (for bacterial toxins and ATP) or internalization of particulate activators by phagocytosis (Cassel et al., 2009). Extracellular ATP (eATP) or bacterial toxins lead to K^+^ efflux and pore formation (Pelegrin and Surprenant, 2007). Phagocytosis of particles including silica, alum, fibrillar amyloid-β protein, or MSU was shown to result in lysosomal destabilization/permeabilization with release of the endosomal–lysosomal protease cathepsin B into the cytoplasm (Halle et al., 2008) and/or to mediate K^+^ efflux and reactive oxygen species (ROS) driven activation. Recently, we and others have described mechanistic links between ATP- and particle-mediated inflammasome activation pathways (Riteau et al., 2012). Here we review the role of active ATP release and purinergic signaling in NLRP3 inflammasome activation.

## ATP AND PURINERGIC SIGNALING

Adenosine triphosphate signaling is emerging as an important mechanism to control various cell functions (Burnstock, 2006; Praetorius and Leipziger, 2009). Cellular stimulation triggers ATP release and subsequently activation of purinergic receptors at the cell surface (autocrine activation) and/or on adjacent cells (paracrine activation), thereby regulating or modulating cellular functions in immunity. Although essentially all cells are able to release nucleotides, the mechanisms underlying nucleotides release by epithelial, endothelial, or other non-excitable cells are poorly understood (Lazarowski et al., 2003; Praetorius and Leipziger, 2009). After release, eATP interacts with specific purinergic receptors or is degraded via different ecto-ATPases to ADP and AMP and then to adenosine. ATP or its metabolites are able to signal through different purinergic receptors (P2X, P2Y, or adenosine P1 receptors; Yegutkin, 2008). In pathological conditions, high levels of ATP are passively released from necrotic cells and act as a pro-inflammatory danger signal, activating the NLRP3 inflammasome through binding to the ionotropic P2X7 receptor (P2X7R; Iyer et al., 2009).

## NLRP3 INFLAMMASOME ACTIVATION: ONE OR TWO STEP SIGNAL?

During NLRP3 inflammasome activation, a two-signal model has been proposed to explain the regulation of IL-1β production by macrophages, dendritic cells, or microglial cells. After a first signal induced by LPS triggers accumulation of pro-IL-1β, exposure to high concentrations of eATP (5 mM) acts as a powerful second signal to elicit the processing of pro-IL-1β into mature IL-1β in murine (Perregaux and Gabel, 1994) and human macrophages (Ataman-Onal et al., 2006) via P2X7R signaling (Di Virgilio, 2007). In contrast, primary human monocytes were shown to require only one signal because LPS alone was sufficient to induce secretion of mature IL-1β, with exogenous ATP acting to further accelerate the LPS-triggered IL-1β processing and secretion (Ferrari et al., 2006; Netea et al., 2009). In addition, primary stimulation of human monocytes with several other PAMPs and one DAMP was sufficient to provide both the first and the second signals via a mechanism involving active release of endogenous ATP to the extracellular environment with consequent activation of the P2X7R in an autocrine loop; this allows the triggering of mature IL-1β secretion in a one step model of inflammasome activation (Piccini et al., 2008). In these studies, eATP and mature IL-1β were measured in the absence/presence of either, P2X7R pharmacologic inhibitors, or apyrase, an ATP/ADP degrading enzyme, to demonstrate the roles of ATP release and purinergic signaling in secretion of mature IL-1β. Moreover, an inhibitor of ATP degradation ARL67156 greatly increased both eATP and IL-1β contents, further supporting the role of endogenously released ATP in secretion of mature IL-1β. These studies demonstrated that LPS or MSU can trigger ATP release from stimulated cells, pointing to fundamental roles for ATP and/or its metabolites as important molecules that mediate the NLRP3 inflammasome activation responses to PAMPs or DAMPS.

Surprisingly, two other recent studies have implicated purinergic signaling but not ATP release in NLRP3 inflammasome activation by suggesting that certain non-nucleotide inflammasome activators may interact directly with purinergic receptors. First, it was shown that soluble biglycan, an ubiquitous leucine-rich repeat proteoglycan of the extracellular matrix, acts as an endogenous danger signal that activates the NLRP3 inflammasome and the release of IL-1β without additional co-stimulatory factors (e.g., exogenous ATP) in murine primary peritoneal macrophages (Babelova et al., 2009). By signaling through TLR2/4, biglycan stimulated the expression of NLRP3 and pro-IL-1β mRNA. Biglycan-induced inflammasome activation was completely inhibited by oxidized-ATP (oATP), which broadly inhibits several P2X and P2Y receptors, and partially inhibited by KN-62, a selective inhibitor of P2X7R, demonstrating the involvement of the P2 purinergic receptors. Moreover, co-stimulation of the biglycan-treated cells with ATP further increased IL-1β secretion. Nevertheless, the authors claimed direct activation of the NLRP3 inflammasome by biglycan and excluded an autocrine role for ATP because biglycan did not affect the secretion of ATP. They proposed that the interaction of biglycan with both TLR2/4 and purinergic P2X4/P2X7Rs induces receptor cooperativity and NLRP3 inflammasome activation (Babelova et al., 2009).

Another study characterized inflammasome responses to serum amyloid A (SAA), an acute-phase protein which undergoes up to a 1000-fold increase in serum levels during inflammation, and which has a pathogenic role in amyloid A-type amyloidosis. SAA provides a signal for both the induction of pro-IL-1β expression and NLRP3 inflammasome activation, resulting in secretion of IL-1β without fibril formation and lysosomal destabilization in human and mouse macrophages (Niemi et al., 2011). Blocking TLR2 and TLR4 attenuated SAA-induced expression of IL-1β, whereas inhibition of caspase-1 or P2X7R by oATP or KN-62 abrogated the release of mature IL-1β. However apyrase treatment did not diminish SAA-mediated IL-1β release and no increase in ATP levels was observed in response to SAA. Thus, the authors proposed that SAA-induced inflammasome activation is mediated by a direct interaction between SAA and P2X7R and that it is not associated with the release of ATP or ADP (Niemi et al., 2011). In conclusion, whether a one- or two-step model of inflammasome activation is involved, ATP and/or purinergic signaling seem to play key roles in NLRP3 inflammasome activation by PAMPs or DAMPs.

## A ROLE FOR ATP IN PARTICLE-INDUCED NLRP3 INFLAMMASOME ACTIVATION

**FIGURE 1:**
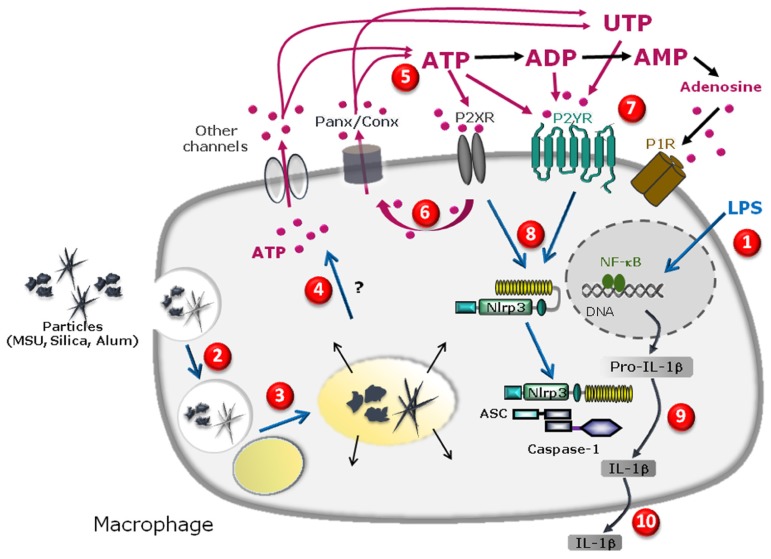
**Schematic diagram illustrating the specific cascade and signaling pathway in LPS-primed macrophages stimulated with MSU, silica, or alum salt crystals.** LPS priming induces transcription of pro-IL-1β gene and other genes in the nucleus upon activation of the transcription factor NFkB and subsequent production of pro-IL-1β protein in the cytosol **(1)**. Particle internalization **(2)**, fusion to lysosome **(3)** and further phagolysosome destabilization may lead to cathepsin leakage **(4)** which precedes pannexin/connexin (Panx/Conx) hemichannel and purinergic signaling-dependent intracellular ATP release **(5)**. Extracellular ATP may act through P2X7 receptor to amplify ATP release in a P2X7 receptor-dependent way **(6)**. ATP, UTP, or their derived degradation products such as ADP, UDP, and adenosine, generated by ecto-endonucleases, may act through an autocrine loop on other purinergic receptor P2X, P2Y, and/or P1 receptors **(7)** leading to NLRP3 receptor activation **(8)**. This allows inflammasome complex formation and maturation of pro-IL-1β to IL-1β production **(9)** and IL-1β secretion **(10)**.

In a recent study, we showed that uric acid, silica, or alum particles induce the active release of intracellular ATP from human macrophage to extracellular compartments via mechanisms that depend on purinergic signaling and connexin/pannexin channels (Riteau et al., 2012). We observed a strong correlation between ATP release and secretion of mature IL-1β after stimulation of phorbol myristate acetate (PMA)-primed THP1 human macrophages. In presence of the first signal (PMA), MSU, silica, or alum salt crystals acted as second signal triggers leading to mature IL-1β production by these human myeloid cells via ATP release and subsequent purinergic signaling. Importantly, allopurinol crystals, which do not elicit NLRP3 inflammasome activation (Ataman-Onal et al., 2006) were also unable to trigger ATP release. In addition, exposure of LPS-primed murine macrophages to the different crystalline stimuli also lead to maturation of IL-1β via pathways dependent on autocrine purinergic signaling loops involving multiple purinergic receptor subtypes (Riteau et al., 2012). Moreover, crystal-induced secretion of IL-1β was abrogated by apyrase to further confirm the role of ATP and autocrine purinergic signaling in inflammasome activation. Nevertheless, P2X7R deficiency in murine macrophages did not change the ability of these cells to secrete IL-1β even though high and non-specific concentrations of the P2X7R inhibitor A740003 severely impaired cytokine production. This suggested the involvement of P2 receptor subtypes in addition to P2X7R. Indeed, several other studies have reported that blocking or deleting P2X7R did not affect MSU- (Ataman-Onal et al., 2006), silica- (Iyer et al., 2009), or alum- (Eisenbarth et al., 2008) induced IL-1β production, thereby implicating roles for other purinergic receptors in non-pathologic conditions. Previous reports have indicated that not only ATP but other nucleotides such as ADP, UTP, or UDP are released into the extracellular space of mechanically stressed cells, in particular endothelial and epithelial cells (Di Virgilio et al., 2001; Lazarowski et al., 2003). These compounds may act on different purinergic receptors to generate a finely tuned response. Our data strongly suggest that members of the two major purinergic receptor families – P2X and P2Y – are involved because ADP and UTP act only on P2Y-family receptors. Thus, our study provides a novel link between the particle internalization- and membrane permeabilization-models of NLRP3 inflammasome activation. Indeed, prior to this analysis, particulate molecules and eATP were considered as two completely independent stimuli for NLRP3 inflammasome activation. The model presented in **Figure 1** summarizes these results and our model.

In another recent report, the P2Y6 receptor was identified as an essential mediator for MSU-induced inflammation in the human THP1 monocyte/macrophage cell line because the specific P2Y6 antagonist MRS2578 completely inhibited MSU-induced IL-1β production (Uratsuji et al., 2012). Because P2Y6 receptors are known to be coupled to the activation of phospholipase C (PLC; Abbracchio et al., 2006), a PLC inhibitor, U-73122, was also tested and shown to suppress the MSU-induced IL-1β production by THP1 cells. These results demonstrate that the P2Y6–PLC signaling pathway mediates MSU-induced inflammatory responses in human monocytes (Uratsuji et al., 2012) and thus point to the involvement of P2Y receptors in particle-induced NLRP3 inflammasome activation.

Interestingly, ATP is a potential mediator of neuroinflammation and an extracellular signaling molecule between neurons and glial cells (Abbracchio et al., 2006). Multiple P2X and P2Y receptor subtypes are expressed by astrocytes, oligodendrocytes, and microglia (James and Butt, 2002). Thus, microglial cells are a good model to investigate physiological functions of purinergic receptors in the immune system. First, eATP was shown to cause a large release of IL-1β from microglial cell lines and from freshly isolated microglial cells by activating the P2X7R (Di Virgilio et al., 1996). Second, stimulation of microglia cells through TLRs has been proposed to induce the release of endogenous ATP acting in an autocrine manner to activate the ion channel P2X7 (Ferrari et al., 1997). Release of ATP and other nucleotides seems to modulate microglial responses via P2Y and P2X receptors, with the P2X7 subtype standing out for its known pro-inflammatory activity and for its up-regulation in both a transgenic mouse model of Alzheimer’s disease and in the brains from Alzheimer’s disease patients (Parvathenani et al., 2003; McLarnon et al., 2006). Moreover, another particulate NLRP3 activator, amyloid-β protein aggregates was shown to promote IL-1β release through P2X7R-mediated ATP release and therefore to activate the NLRP3 inflammasome in microglia (Sanz et al., 2009).

In summary, four different particulate activators, MSU, silica, alum crystals, and amyloid-β protein aggregates have been shown to activate the NLRP3 inflammasome through mechanisms involving ATP release and autocrine purinergic signaling. Based on the use of pharmacological inhibitors, a role for P2X7R has often been reported. However, the uncertain selectivity of P2X7R antagonists (which can vary with concentration and cell type) may yield questionable or equivocal conclusions. Our recent study used pharmacological agonists, antagonists, and P2R subtype-deficient mice to suggest that multiple purinergic signaling pathways are involved in NLRP3 inflammasome regulation through activation by ATP, ADP, UTP, UDP, and/or adenosine.

## A ROLE FOR ATP RELEASE IN CELL DEATH-INDUCED INFLAMMASOME ACTIVATION

**FIGURE 2:**
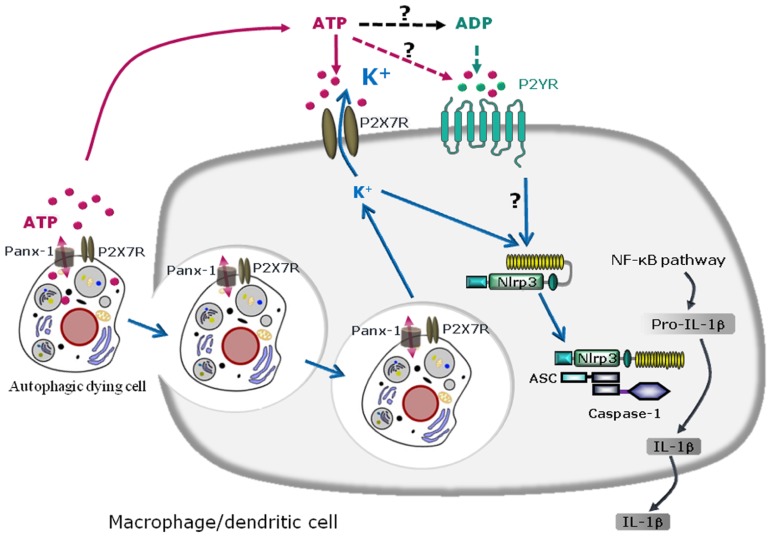
**Schematic diagram illustrating the mechanisms of NLRP3 inflammasome activation by autophagic dying cells through ATP leakage and purinergic signaling in murine macrophages or dendritic cells**. Autophagic dying cells release ATP through pannexin-1 (Panx-1) hemichannel resulting in activation of the purinergic receptor P2X7R on macrophages or dendritic cells. Phagocytosis of autophagic dying cells and K^+^ efflux are required for NLRP3 inflammasome activation in macrophages or dendritic cells. Since extracellular ATP is rapidly degraded in ADP, AMP, and adenosine, ATP metabolites could also act through other purinergic receptors and in particular, ATP and ADP could signal through P2YR. Purinergic signaling and K^+^ efflux result in inflammasome assembly and caspase-1 activation leading to maturation of pro-IL-1β in IL-1β and IL-1β secretion.

Cells which die in response to non-developmentally programmed cues, such as the necrosis produced by pressure disruption, hypoxic injury, or complement-mediated damage, are potent activators of the innate immune system and can promote sterile inflammation through sensing by the NLRP3 inflammasome that results in the subsequent release of the pro-inflammatory cytokine IL-1β. This activation may be triggered in part by ATP produced by mitochondria and released from damaged cells (Iyer et al., 2009). Cells which die as part of physiological responses, such as apoptotic or autophagic cells, are removed from tissues to prevent immune reactions and maintain tissue homeostasis. Although apoptotic cells have anti-inflammatory properties due to their surface exposure of anti-inflammatory molecules (Fadok et al., 1998; Cvetanovic et al., 2006), cells dying via autophagy can trigger pro-inflammatory responses through the release of danger signals that drive NLRP3 inflammasome activation (Petrovski et al., 2007b; Ghiringhelli et al., 2009; Michaud et al., 2011). Since the first description of autophagy in 1966 (De Duve and Wattiaux, 1966), numerous studies have described autophagy as a survival mechanism response to poor nutritional conditions (Gozuacik and Kimchi, 2007; Maiuri et al., 2007). However, it is now clear that autophagy has a dual role (Codogno and Meijer, 2005): under certain circumstances, autophagy constitutes a stress adaptation that avoids cell death (and suppresses apoptosis) by degradation of long-lived proteins and damaged organelles through the autophago-lysosomal pathway, whereas in other cellular settings, it constitutes an alternative cell-death pathway (Petrovski et al., 2007a). Cell dying through autophagy were shown recently to induce a pro-inflammatory response in human macrophages (Petrovski et al., 2007b). Moreover, phagocytosis of human cancer cells dying through autophagy was shown to trigger NLRP3 inflammasome activation and maturation of IL-1β in human macrophages via an ATP-dependent mechanism (Petrovski et al., 2011). Indeed, multiple manipulations including blockade of K^+^ efflux during phagocytosis, incubation in the presence of apyrase, addition of P2X7R antagonist, or silencing NLRP3 protein expression, all acted to inhibit this IL-1β secretion response (Petrovski et al., 2011). Moreover, phagocytosis of murine cells, dying via autophagy by mouse macrophages was found to activate the NLRP3 inflammasome in the engulfing macrophages (Ayna et al., 2012). Studying the mechanism of inflammation illuminated roles for ATP release via pannexin-1 channels in the autophagic dying cells, phagocytosis of autophagic dying cells, P2X7R activation and subsequent K^+^ efflux in macrophages as obligatory steps for NLRP3 inflammasome activation in this model (Ayna et al., 2012). Together, these studies have demonstrated that ATP release is required for NLRP3 inflammasome activation in both human and murine macrophages. Some mechanistic details may differ in these models; during phagocytosis of human autophagic dying cells, ATP is released by macrophages and acts on macrophage purinergic receptors in an autocrine loop (Petrovski et al., 2007b), whereas during engulfment of murine autophagic dying cells, ATP is released by autophagic dying cells and acts on macrophages in an paracrine loop (Ayna et al., 2012). Importantly, autophagic death was reported to contribute to making apoptotic cancer cells immunogenic (Michaud et al., 2011; Petrovski et al., 2011) and thereby capable of activating the inflammasome in dendritic cells (Ghiringhelli et al., 2009). These authors first showed in a murine model that treatment of cancer cells with anticancer chemicals (such as oxaliplatin and mitoxantrone) causes immunogenic cancer cell death (Ghiringhelli et al., 2009). ATP released from dying tumor cells was shown to activate P2X7R signaling in dendritic cells, leading to inflammasome activation and further IL-1β secretion (Ghiringhelli et al., 2009). These authors also demonstrated that oxaliplatin- or mitoxantrone-treated tumor cells die via autophagy which induces an immunogenic response *in vivo* by recruiting dendritic cells and T cells into the tumor through the release of ATP into the extracellular fluid (Michaud et al., 2011). They subsequently reported that autophagy is essential for the immunogenic release of ATP from dying cells (Michaud et al., 2011). Furthermore, such an immunogenic anti-tumor response could also be elicited when autophagy and cell death was induced by cytokine depletion. The mechanisms for the NLRP3 activation triggered through murine cells dying via autophagy are summarized in **Figure 2**.

In conclusion, an increasing body of evidence suggests that ATP and/or purinergic signaling are cornerstone regulators of NLRP3 inflammasome activation in many, but not all, biological contexts wherein the purinergic pathways do not exclude the existence of other mechanisms. First, a high amount of passive ATP release from necrotic cells activates the inflammasome through the P2X7R. Second, PAMP recognition and signaling through their receptors trigger active ATP release in some cell types such as human monocytes. Third, phagocytosis of several inflammasome activators following by ATP release appears to be common pathway for activating the NLRP3 inflammasome. Fourth, ATP leakage from autophagic dying cells and the engulfment of these cells by macrophages trigger immunity through NLRP3 inflammasome activation. It will be important to identify the events linking, on the one hand, phagocytosis of particles or autophagic dying cells to ATP release by macrophages, and on the other hand, ATP leakage and engulfment of autophagic dying cells to NLRP3 activation. Finally, the events triggering NLRP3 inflammasome assembly and activation downstream of purinergic signaling are unknown. Another important issue is that ATP and P2X7R are not the only purinergic “players” in this response because different nucleotide metabolites such as ADP, UTP, UDP, and adenosine, and other members of the purinergic receptor family, i.e., the P2X, P2Y, and P1 receptors may contribute through complex purinergic signaling networks. Better understanding of these mechanisms will facilitate identification of new targets for inflammatory diseases and improve our understanding of the immune response to cancer.

## Conflict of Interest Statement

The authors declare that the research was conducted in the absence of any commercial or financial relationships that could be construed as a potential conflict of interest.
